# Modulating Charge Mobility in Microwave Synthesized Ti-doped ZnS Nanoparticles for Potential Photoanode Applications

**DOI:** 10.3390/nano13010077

**Published:** 2022-12-23

**Authors:** Mpho W. Maswanganye, Guy L. Kabongo, Mokhotjwa S. Dhlamini

**Affiliations:** Department of Physics, College of Science Engineering and Technology, University of South Africa, Private Bag X6, Florida, Science Campus, Christiaan de Wet and Pioneer Avenue, Florida Park, Johannesburg 1710, South Africa

**Keywords:** ZnS nanoparticles, microwave irradiation, Williamson–Hall method, microstrain, electrochemical impedance spectroscopy

## Abstract

Doping ZnS nanoparticles with different metal and/or non-metal ions is one of the ways to improve their properties. That is because dopants introduce strain into the lattice of the ZnS nanoparticles. The influence of Ti on the ZnS nanoparticles was investigated on the structural properties, optical properties, and also electrical impedance spectroscopy (EIS). The presence of Ti in the crystal lattice of the ZnS introduced strain into the crystal structure, hence causing a lattice expansion and reducing the crystallite sizes of the ZnS nanoparticles. Ti doping was observed to increase the energy band gap of ZnS nanoparticles and also reduce the charge carrier recombination. Doping Ti into ZnS was observed to decrease the charge transfer resistance of ZnS nanoparticles with an increase in dopant concentration indicating an improved charge transfer mobility owing to the presence of strain in the crystal lattice.

## 1. Introduction

Dye-Sensitized Solar Cells (DSSCs) are appealing among solution-processable solar technologies because they are manufactured from low-cost substances that do not require high purification, potentially lowering the cost of photovoltaic electricity generation [1,2]. Over the last two decades, there has been significant academic and, increasingly, commercial interest in this technology, which has been fuelled by many significant advances in the device’s material components [3,4]. Similar to material advancements, significant progress has been made in understanding the science underpinning device performance. Solar cell action is triggered in these devices upon irradiation of light by excitonic electron-hole pair formation and dissociation, resulting in charge separation at the nano-interface. 

ZnS and metal-doped ZnS nanostructure materials have been extensively explored, beginning with synthetic procedures and progressing to applications, particularly solar cells [5]. One potential application for ZnS was as an anode material or a buffer layer for solar cells. ZnS has acquired a lot of attention since its properties are similar to those of classic semiconductors such as titanium dioxide (TiO_2_) or zinc oxide (ZnO). Wide band gap semiconductors were assigned to be the mesoporous anode in dye-sensitized solar cells (DSSCs), however, they might be employed as a buffer layer in quantum dot-sensitive solar cells [5,6]. ZnS is an important member belonging to the II–VI group of semiconductors. It is a commercially significant semiconductor having a band gap of 3.68 eV at room temperature that falls in the ultraviolet region of the electromagnetic spectrum [7]. Its optical properties might be tuned because of the size-dependent variety of the band gap energy. The quantum confinement impacts high surface area and nontoxicity possessed by ZnS nanoparticles justify their use as potential candidates for applications such as optoelectronic devices, buffer layer in sun-light oriented photovoltaic, phosphors, UV photodetectors, light emitting diodes, and photocatalysis [8,9]. 

ZnS nanoparticles can be considered the best host for adjusting the optimal band gap engineering for the electric applications of effective optoelectronic devices [10,11]. Various techniques including Microwave [12], Hydrothermal [13], solid state [14], and sol-gel [15] have been used to synthesize ZnS nanostructured materials. Nanostructured materials are characterized as materials comprising hundreds to thousands of atoms. This field of material science has been created quickly and drawn into broad studies during the past twenty years because of its ability to deliver nanostructured materials with unique structure, and optical and electronic properties that originate from their large surface-to-volume proportion [16]. The nanoparticle’s surface states can be adjusted by the variety of the nanoparticle’s grain size through the doping process [17]. The interfacial surface state defects in the nanoparticles grains significantly affect numerous physical properties connected with the optical excitation of charge carriers to higher energy levels inside the material or charge carrier transport between the nanoparticles grains because of electrical field excitation. The preparation of the nanoparticles through a chemical precipitation process is represented by different parameters such as reactant molarity in the solvent, the temperature during the chemical reaction, and the presence of chelating agents to control the nanoparticle’s size. Additionally, the incorporation of extraneous cations in the nanoparticle’s host lattice would give further controlling parameters to the nanoparticle’s development after the nucleation cycle. Consequently, the nanoparticle’s size and the related grain boundaries would be altered. The impact of doping process on nanoparticles development during the chemical precipitation step is depicted either by the reversal of grain boundary development [18] or grain boundary inhabitation brought about by the pinning force applied to the grain surface as per Zener’s drag phenomenon [19]. Doping different elements into materials brings about a lattice mismatch caused by the strain effect. The strain effect occurs in the core/shell structure, where the core and shell have different lattice parameters, and in solid solution alloys, where atoms of one metal are absorbed into the lattice of another metal. Recently, microstrain, or localized lattice strain, which is strongly connected with structural defects such as grain boundaries, has been rationalized to improve electrocatalytic activity and stability via distinct processes with the aforementioned strain effect [20]. There is a great need to understand the effect of strain in order to be able to improve the physicochemical properties of materials. Understanding the effect of stain can open channels for ZnS in fields such as photoanode applications. In this work, we have synthesized undoped ZnS and Ti-doped ZnS nanoparticles by microwave irradiation method in order to understand and improve their structural, optical, and EIS properties.

## 2. Experimental Details

### 2.1. Sample Preparation 

The undoped-ZnS nanoparticles and Ti-doped ZnS nanopowders were synthesized by microwave method. Zinc acetate dehydrate [Zn(CH_3_CO_2_)_2_·2H_2_O], thioacetamide [C_2_H_3_SNH_2_], titanium acetate [Ti(C_2_H_3_O_2_)_4_] (all purity of 98.0% from Sigma-Aldrich, Modderfontein, Gauteng, South Africa) were used as precursor materials. Deionized water was used as the only solvent. 

Typically, for the preparation of the undoped-ZnS nanoparticles, 0.005 mol of Zn(CH_3_COO)_2_·2H_2_O (solution A) and 0.005 mol of C_2_H_3_SNH_2_ (solution B) were dissolved and stirred in 10 mL deionized water for 10 min. After continuous magnetic stirring for 10 min, solutions A and B were mixed and stirred for another 10 min. For the preparation of Ti-ZnS nanopowder samples, 0.5, 0.75, and 1.00% concentration by mole of Ti(C_2_H_3_O_2_)_4_ (solution C) were dissolved and stirred in 10 mL deionized water for 10 min. After continuous magnetic stirring for 10 min, solution C was mixed together with solutions A and B and stirred for 10 min. The final solutions were then poured into a glass tube and then placed inside a microwave. The microwave temperature was allowed to ramp to 150 °C for 30 min where then the reaction took place for 30 min at 150 °C, with the temperature automatically dropping to room temperature after the reaction. The products were collected and washed in a centrifuge with deionized water and ethanol several times at 4000 rpm for 10 min. After washing, the samples were then dried at 60 °C overnight. 

### 2.2. Characterization 

The X-ray diffraction (XRD, Rigaku Corporation, Tokyo, Japan) pattern was performed with a Rigaku D/max-2500 X-ray diffractometer (Cu-Κα radiation, λ = 1.5406 Å) from 20–70 degrees. The morphology was characterized using JEOL JSM-7800F (JOEL Ltd, Letchworth, UK) field emission scanning electron microscopy (FE-SEM) coupled with energy dispersive X-Ray spectroscopy (EDS), the samples were first coated with carbon before measurements were performed to avoid the samples from charging. The diffuse reflectance properties of the materials were studied using the Shimadzu UV-2600i spectrophotometer (Kyoto, Japan) in the range of 250–800 nm. The vibrational studies were conducted using Perkin Elmer Fourier transform infrared spectroscopy (FTIR) in the range 400–4000 cm^−1^. The room temperature photoluminescence (PL) emission spectra were recorded with a Fluorolog Horiba Jobin-Yvon (Miyanohigashi-cho, Kisshoin Minami-Ku Kyoto, Japan) equipped with 450W xenon lamp as an excitation source. 

A three-electrode configuration system Autolab PGSTAT302N (Metrohm AG, Herisau, Switzerland) electrochemical working station was employed to do the electrochemical impedance spectroscopy measurements in a 1 M electrolyte solution of Na_2_SO_4_. The undoped ZnS and the Ti-doped ZnS nanoparticles were added with a polyvinylidene difluoride (PVDF). A few drops of DMSO were later added to the mixture in order to form a precipitate. The precipitate was then ultrasonicated for 30 min. The precipitate was then drop-coated onto an ITO conductive glass substrate of 2.5 cm × 1.8 cm. The glass substrates were then dried in an oven at 60 °C for 60 min. Ag/AgCl chloride was used as a reference electrode and Pt was used as a counter electrode connected to an Autolab instrument. The working electrode was placed upright with the basel part facing the source of light at an 8 cm distance. A solar simulator, with a xenon lamp, produced visible light intensity of 100 mW cm^−2^. The EIS measurements were done at a frequency of 0.1 Hz–100 kHz at an amplitude of 10 mV.

## 3. Results and Discussions

### 3.1. X-ray Diffraction

**Figure 1:**
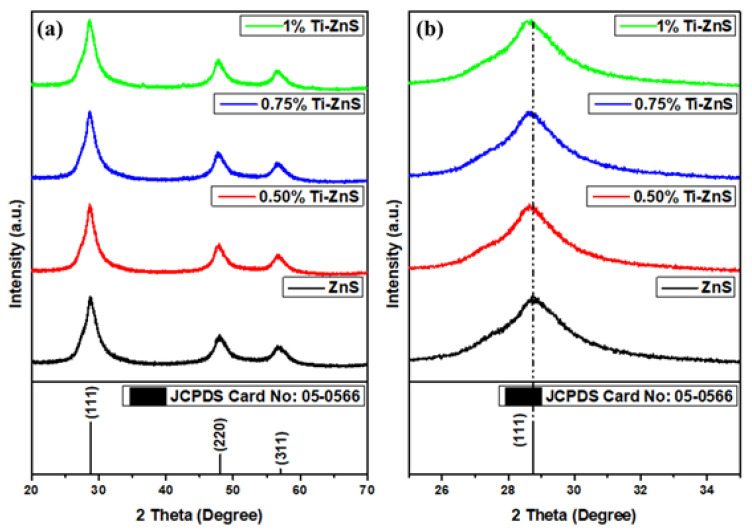
(**a**) XRD patterns of undoped ZnS and Ti-doped ZnS nanopowder samples at different concentrations, (**b**) peak shift at (111).

Figure 1a shows the XRD patterns of the undoped ZnS and Ti-doped ZnS nanopowder samples at 0.50, 0.75, and 1.00 % mol concentration. All the prepared samples were found to be of ZnS cubic structure (JCPDS No.05-0566), as shown in Figure 1b. There were no impurities observed for all the prepared samples. A peak shift towards lower 2-theta angles has been observed with an increase in dopant concentration as indicated in Figure 1b, a close analysis of the XRD patterns uncovers that all the XRD peaks shift towards lower angles which indicates the presence of Ti in the ZnS crystal structure [21,22]. The shifting towards lower angles shows that there is a lattice expansion in the Ti-doped ZnS nanopowder samples. Similar results were reported by A. Jafari-Rad and H. Kafashan [23] with the prediction that Ti substituted for Zn causing the lattice expansion of the ZnS crystal structure. 

The lattice parameters *a = b = c* for the prepared nanopowder samples were determined using the following equations:

(1) $$2d_{hkl}\sin\theta =n\lambda_{CuK\propto }$$

(2) $$a=d_{hkl}{\left(h^{2}+k^{2}+l^{2}\right)}^{1/2}$$

where $\lambda_{CuK\propto }$ is the wavelength of the XRD, *n* is the order of diffraction (for first order n = 1), *d_hkl_* is the interplanar spacing and *h, k, l* is the Miller indices. The estimated lattice constant *a* for all the samples was found to be comparable with the reported values of the bulk ZnS (JCPDS No.05-0566). As observed in Table 1, the lattice constants are increasing with an increase in Ti concentration. The volume of a unit cell of the prepared samples has also been determined using the following formula:

(3) $$V=a^{3}$$

The volumes of the unit cell for the Ti-doped ZnS nanoparticles were found to increase with an increase in dopant concentration, results are shown in Table 1. This increase indicates that Ti has substituted Zn in the lattice causing the crystal structure to expand. 

**Figure 2:**
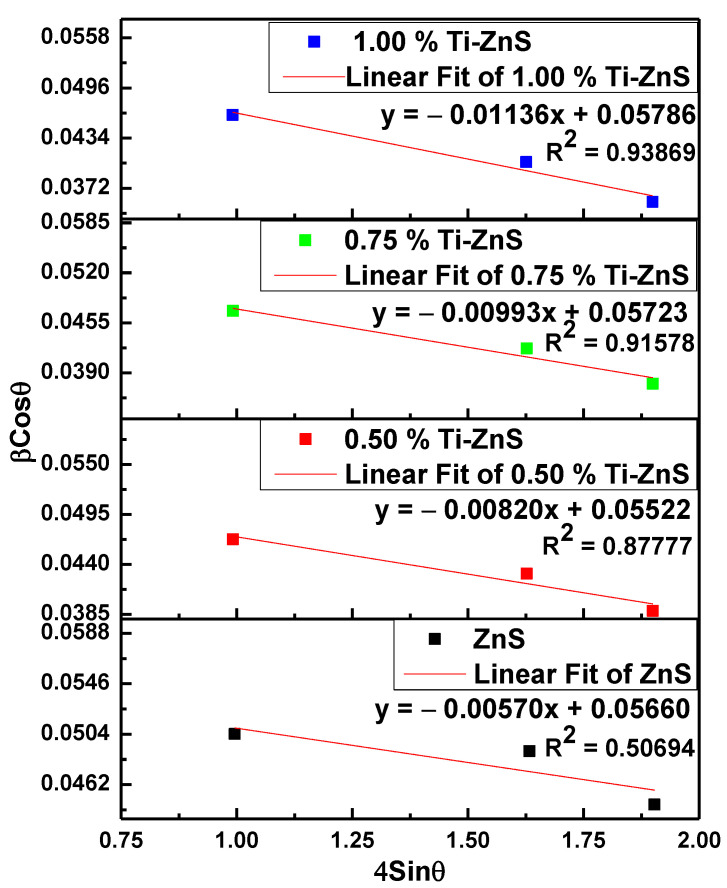
Williamson–Hall plot of undoped ZnS and Ti-doped ZnS nanopowder samples at different concentrations.

The micro-strain (*ε*) and average crystallite size (*D*) are estimated using the Williamson–Hall (W–H) technique given by the equation [24]:

(4) $$\beta_{hkl}\cos\theta =4\varepsilon \sin\theta +\frac{0.9\lambda_{CuK\propto }}{D}$$

where *λ_CuKα_* = 1.5406 Å, *β_hkl_* is the full width at half maximum intensity (FWHM) of the XRD peaks in radians and *θ* is the Bragg’s angle of the diffraction peak. The plot of $\beta_{hkl}\cos\theta$ versus 4sinθ can give strain instigated on the particles and crystallite size which are proportional to the slope of the plot and y-intercept, respectively. Figure 2 shows the W–H plots of the ZnO nanoparticle samples. The calculated values of the average crystallite sizes are shown in Table 1. 

The average crystallite sizes have been observed to decrease with an increase in dopant concentration. The micro-stains for the Ti-ZnS nanopowder samples were found to be negative, as indicated in Figure 2, with the negative sign indicating that the ZnS doped samples are under compressive strain [25]. The structural parameters dislocation density (*δ*), and stacking fault (*SF*) of cubic ZnS nanoparticles were calculated using the equations [24]:

(5) $$\delta =\frac{1}{D^{2}}$$

(6) SF=[2π245(3tanθ)12]β

The lattice defect *δ* was observed to increase with an increase in dopant concentration. This increase in *δ* might be due to the presence of doping atoms at grain boundaries and indicating poor lattice quality with an increase in dopant concentration. SF showed a decreasing trend with increasing Ti doping content, which may be due to the improvement of crystallinity.

### 3.2. Scanning Electron Microscopy (SEM) and EDS

**Figure 3:**
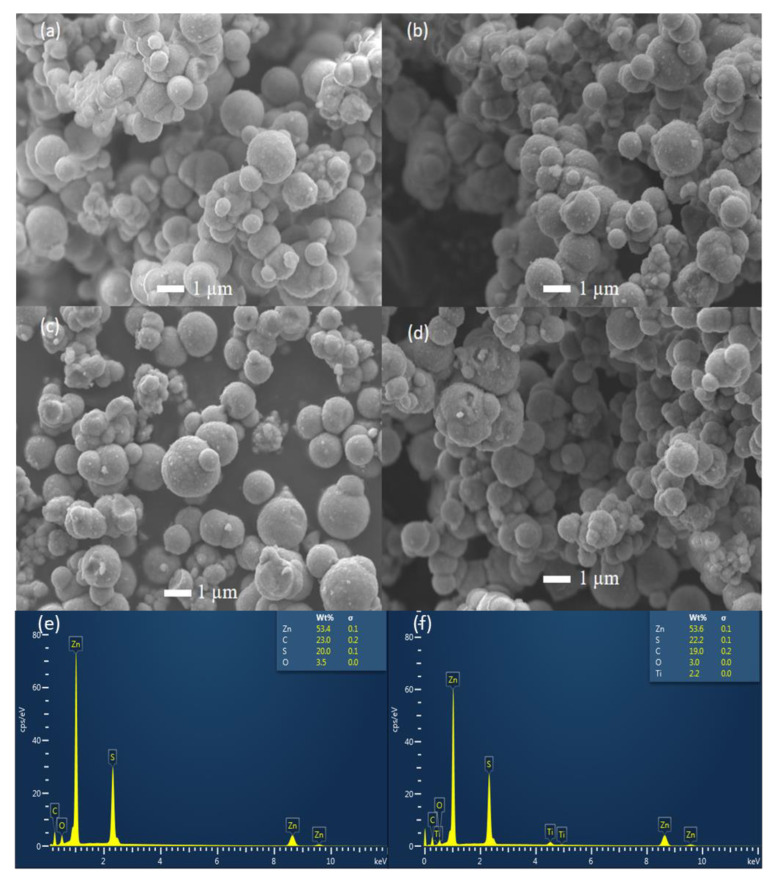
SEM micrographs of (**a**) undoped ZnS nanoparticles, (**b**) 0.50% Ti-doped ZnS nanoparticles, (**c**) 0.75% Ti-doped ZnS nanoparticles, (**d**) 1.00% Ti-doped ZnS nanoparticles, EDS analysis for (**e**) undoped ZnS nanoparticles, and (**f**) 1.00% Ti-doped ZnS nanoparticles.

The investigation of the surface morphology of the ZnS-prepared samples was achieved by SEM. The SEM micrographs of the undoped-ZnS and Ti-doped ZnS samples are shown in Figure 3. From Figure 3, it is revealed that the shape of the prepared ZnS nanoparticles samples is spherical for all the samples. The crystallite size is observed to be decreasing with an increase in dopant concentration indicating that restricts the crystallite sizes to grow, demonstrating a compressive strain of the grain boundary of the particles. Figure 3e,f show the EDS results of undoped ZnS and 1.00% Ti-doped ZnS nanoparticles respectively. No impurities have been observed in all the prepared ZnS nanoparticles. Figure 3f indicates that Ti has successfully been incorporated into the ZnS nanoparticles. 

### 3.3. FTIR Spectroscopy

**Figure 4:**
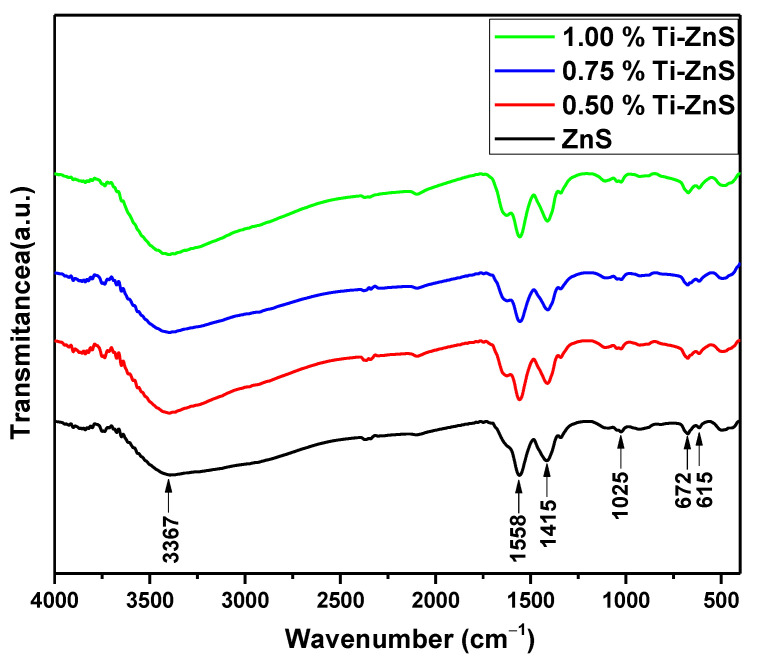
FTIR spectra of undoped ZnS and Ti-doped ZnS nanopowder samples at different concentrations.

FTIR spectroscopy has been widely used to discover the organic species and elemental composition present in the sample and understand the chemical bonds’ nature. Figure 4 shows the FTIR spectra of the Ti-ZnS nanopowder samples prepared through the microwave method. The infrared (IR) mode detected around 3367 cm^−1^ is attributed to the stretching vibration of the O−H group, which might be due to the absorption of H_2_O molecules during the preparation process on the sample surface [26]. The prominent vibrational modes appearing around 1415 cm^−1^ rise to the bending vibrational modes of the H-O−H bond of the H_2_O molecule in the sample. Further, the vibrational peak around 1025 cm^−1^ confirms the formation of microstructure in the samples. For Fe-doped ZnS samples, this peak around 1025 cm^−1^ shifted to the higher wavenumbers, which indicates the successful doping of Ti ions into the Zn sites in the ZnS host system [26,27]. However, the peaks aroused around 672 and 615 cm^−1^ further confirm the Zn−S stretching mode vibrations [27]. The obtained FTIR spectra of the undoped ZnS and Ti-doped ZnS samples are in good agreement with the reported literature.

### 3.4. UV-Vis Spectroscopy

**Figure 5:**
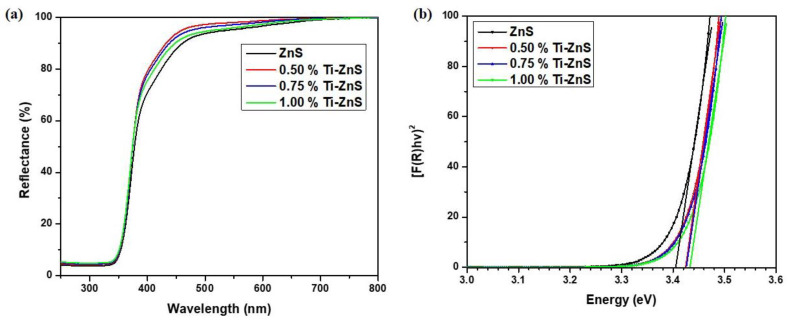
(**a**) UV-Vis spectra, (**b**) plot of [F(R) hν]^2^ vs hν of undoped ZnS and Ti-doped ZnS nanopowder samples at different concentrations.

For contemplating the optical properties of the undoped ZnS and Ti-doped ZnS nanopowder samples, the nature of the band gap was determined utilizing the UV-Vis-NIR spectrophotometer in diffuse reflectance mode at room temperature over a scope of wavelength (250–800 nm). Figure 5a shows the diffuse reflectance spectra of the undoped ZnS and Ti-doped ZnS nanopowder samples. The energy band gap of the prepared samples was estimated using the Kubelka–Munk (K–M) function. This K–M theory describes the behaviour of the light path through a dispersing medium as a function of the scattering (S) and the absorption (*k*) coefficients with *R_∞_* the diffuse reflectance for an infinite sample [28]:

(7) $$F\left(R_{\infty }\right)=\frac{{\left(1-R_{\infty }\right)}^{2}}{2R_{\infty }}=\frac{k}{S}$$

Nonetheless, in a parabolic band structure, the energy band gap and absorption coefficient are connected through Tauc’s equation [29].

(8) $$\left(\alpha h\upsilon \right)=A{\left(h\upsilon -E_{g}\right)}^{m}$$

where α is the absorption coefficient of the sample, *hν* the photon energy, *A* is an energy autonomous constant, *Eg* is the energy band gap, and m is a consistent dependent upon the band gap nature by and generally *m* = 1/2 or 2 for allowed direct and indirect band gap material. Assuming the samples disperses in a completely diffuse way, K–M function can be written in the form of the Tauc’s condition as [29]:

(9) $$F\left(R_{\infty }\right)h\upsilon =B{\left(h\upsilon -E_{g}\right)}^{m}$$

The energy band gaps of all prepared nanopowder samples were estimated from the plots of [F(R) hν]^2^ versus photon energy (hν) by extrapolation of the linear least square fit of [F(R) hν]^2^ to zero [28]. Figure 5b shows the plots [*F(R) hν*]^2^ vs *hν*. The values of estimated energy band gaps of the ZnS nanopowder samples have been listed in Table 2. It can clearly be seen that the energy band gap increases with an increase in dopant concentration. This increase in the energy band gap is due to the decrease in the crystallite size where there is an increase in the surface-to-volume ratio which is attributed to the quantum confinement effect [30,31]. This effect states that the surface potential barrier confines the electrons and holes spatially. The lowest optical transition energy level between the valence and conduction bands is enhanced as a result. This causes the band gap to widen and indirectly links the band gap to crystallite size.

### 3.5. PL Spectroscopy 

**Figure 6:**
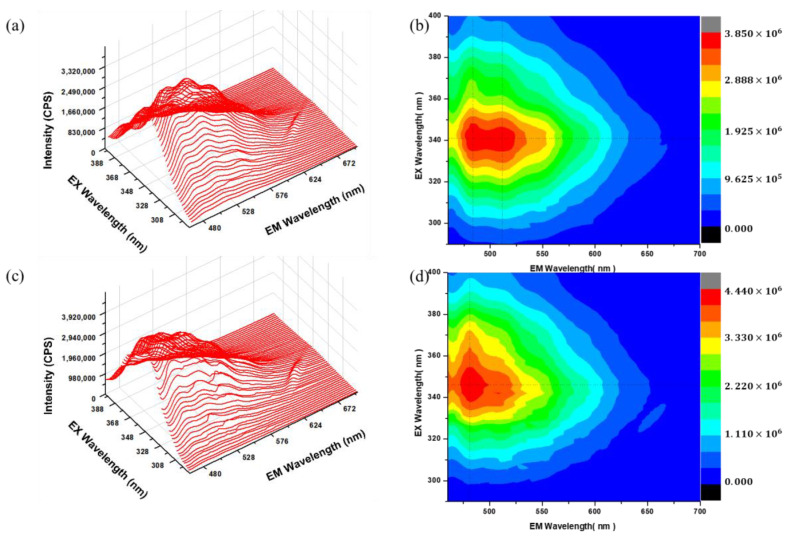
Three-dimensional (3D)-PL spectra of (**a**) undoped ZnS nanopowder, (**b**) Ti-doped ZnS nanopowder 3D) PL/PLE map, (**c**) 3D-PL spectra of Ti-doped ZnS nanopowder, (**d**) Ti-doped ZnS nanopowder 3D PL/PLE map.

The electrons are excited from the valance band to the conduction band using photons with energy equivalent to the band gap. The PL spectra for ZnS are seen in the visible range, which may be caused by crystal point defects. The defect density rises as a result of the high surface-to-volume ratio of nanoparticles at decreasing dimensions. PL measurements were conducted at room temperature to study the effect of dopants on undoped ZnS and Ti-doped ZnS nanoparticles. The PL 3-D spectra’s together with their 3-D contour map are collected as shown in Figure 6a–d. This was done in order to find the best excitation wavelength. As can be observed in Figure 6b the best excitation wavelength is around 340 nm and thus emission measurements were measured using the excitation of 340 nm. 

**Figure 7:**
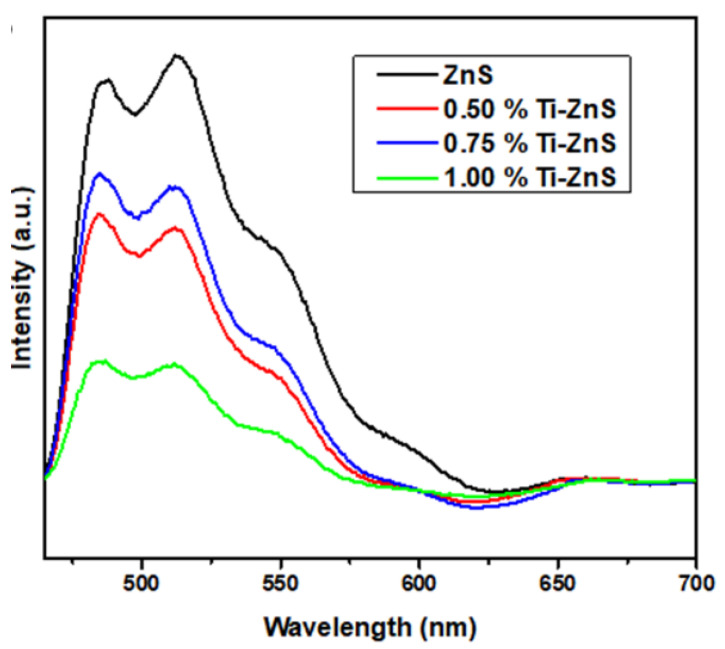
PL spectra of undoped ZnS and Ti-doped ZnS nanopowder samples at different concentrations.

Zinc vacancy (V_Zn_), sulphur vacancy (V_S_), interstitial Zinc (I_Zn_), and interstitial Sulphur (I_S_) are the typical Schottky defects that are frequently found in ZnS [12]. Figure 7 shows the PL spectra of undoped ZnS and Ti-doped ZnS nanoparticles doped at different concentrations. All of the prepared samples have broad and asymmetric spectra, indicating the presence of several contributors to the PL intensity. From Figure 7 four emissions can be observed at around 485 nm, 512 nm, 552 nm, and 594 nm. The peak at 486 nm is attributed to V_s_ [25]. The emission peak at around 512 nm may be assigned to I_Zn_ and is observed to be decreasing with an increase in dopant concentration which may indicate that Ti has substituted for Zn. This is also confirmed in the 3-D contour map of the 1.00% Ti–doped ZnS in Figure 6d. The weak emission peak at 552 nm should be linked with point defects, which is most possibly V_Zn_ [32]. The emission peak visible at longer wavelengths (594 nm) may also be caused by V_Zn_. From Figure 7 we can clearly see that there is a quenching as the concentration is increased. These may indicate that as the dopant concentration increases the charge carrier recombination decreases with the 1.00% Ti-doped ZnS having the lowest intensity [22].

### 3.6. Electrochemical Impedance spectroscopy

**Figure 8:**
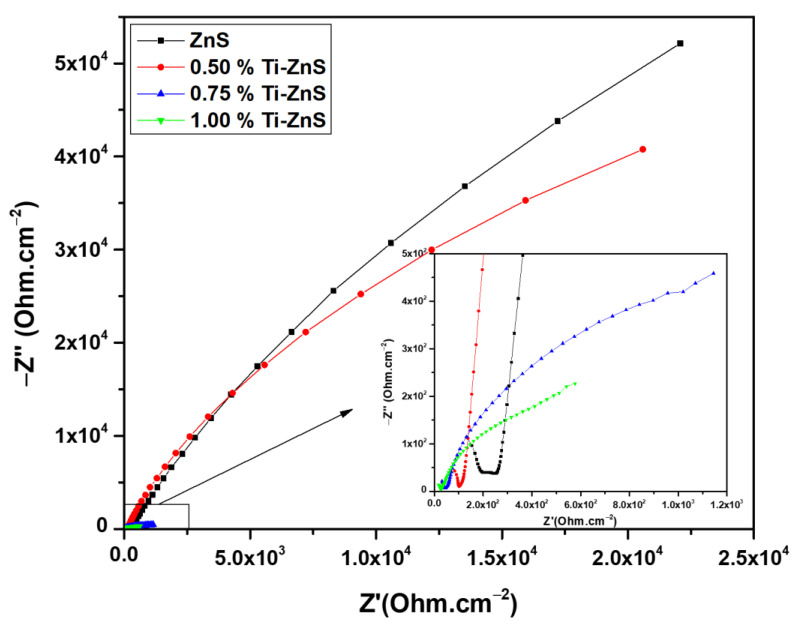
Nyquist plots of undoped ZnS and Ti doped ZnS thin films under illumination.

**Figure 9:**
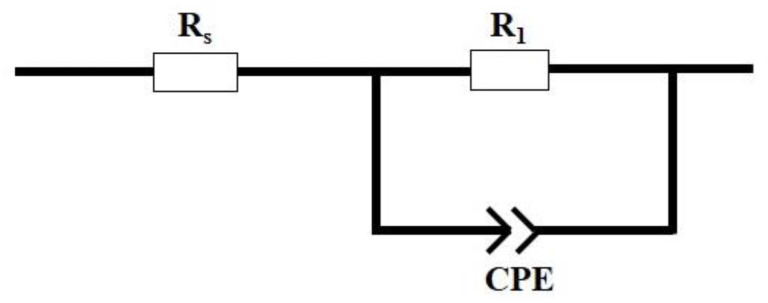
EIS equivalent circuit of the undoped and Ti-doped ZnS.

To investigate the effect of Ti dopant on the charge mobility properties of Ti-doped ZnS film we conducted EIS measurements under illumination (see Figure 8). The best fitting was obtained using the equivalent circuit shown in Figure 9 from the EIS Spectrum Analyser software. The EIS parameters obtained for both undoped ZnS and Ti-doped ZnS thin films are presented in Table 3. The important EIS parameters are R_s_ which denotes the electrolyte resistance, R_1_ denotes the charge transfer resistance, and CPE is the constant phase element.

It can clearly be observed from Figure 8 that the electrical resistance decreases with an increase in dopant concentration under illumination. Table 3 also indicates that the electrical resistance decreases as the concentration of Ti is increased. This indicates that as the concentration increases, the conductivity of the Ti-doped ZnS thin films also increases, showing an improvement in electron flow. This finding confirms that strain induced in ZnS due to Ti doping considerably enhanced the charge mobility in the prepared thin films. Hence, this demonstrates that the present material is promising for application in photoanode fabrication.

## 4. Conclusions

The results in this study demonstrated an improvement in the structural and optical properties, and the EIS measurements of ZnS nanoparticles after being doped with Ti. The lattice parameters and the unit cell volume of Ti-doped ZnS nanoparticles were found to be increasing with an increase in dopant concentration due to the presence of Ti causing strain into the ZnS nanoparticles. The presence of Ti reduced the crystallite size of the ZnS nanoparticles resulting in a higher surface area improving the structural properties of ZnS nanoparticles. The energy band gap was observed to increase with an increase in Ti concentration. Doping ZnS with Ti resulted in some of the defects’ related emissions diminishing with an increase in dopant concentration indicating a decrease in defects and also a decrease in charge carrier recombination. These demonstrate an improvement in optical properties. Finally, EIS measurements indicated a decrease in the materials’ electrical resistance with an increase in dopant concentration indicating an improvement in electron mobility measurements.

## Figures and Tables

**Table 1:** Lattice parameters, d spacing, average unit cell volume (V), average crystallite size (D), micro strain (ε), dislocation density (δ), and stacking Fault (SF).

Sample Name	2θ (Deg.)	d (Å)	a (Å)	Ave. of a (Å)	Ave. of V (Å)^3^	D (nm)	ε (10^−3^)	δ (10^17^ lines/m^2^)	SF
ZnS	28.833	3.094	5.359	5.355	153.564	2.558	−5.700	1.528	0.022
	48.188	1.887	5.337						
	56.827	1.619	5.369						
0.5% Ti-ZnS	28.714	3.106	5.381	5.372	155.004	2.622	−8.200	1.454	0.020
	48.010	1.893	5.356						
	56.713	1.622	5.379						
0.75% Ti-ZnS	28.713	3.107	5.381	5.372	155.026	2.530	−9.930	1.562	0.019
	48.006	1.894	5.356						
	56.711	1.622	5.379						
1.00% Ti-ZnS	28.697	3.108	5.383	5.375	155.242	2.503	−11.360	1.596	0.018
	47.976	1.895	5.360						
	56.405	1.622	5.380						

**Table 2:** Energy band gap.

Sample	*E_g_ (eV)*
ZnS	3.4052
0.5% Ti-ZnS	3.4244
0.75% Ti-ZnS	3.4260
1.00% Ti-ZnS	3.4335

**Table 3:** EIS parameters determined from the equivalent circuit.

Sample	Rs (Ω)	R_1_ (kΩ)	CPE (µF)	n	C (µF)
ZnS	208.23	527.68	26.24	0.84	43.29
0.50% Ti-ZnS	103.76	174.79	29.66	0.88	37.12
0.75% Ti-ZnS	38.38	1.65	387.91	0.56	273.19
1.00% Ti-ZnS	23.34	0.60	500.00	0.58	209.09

## Data Availability

The data presented in this study are available on request from the corresponding authors.
